# How far MS lesion detection and segmentation are integrated into the clinical workflow? A systematic review

**DOI:** 10.1016/j.nicl.2023.103491

**Published:** 2023-08-12

**Authors:** Federico Spagnolo, Adrien Depeursinge, Sabine Schädelin, Aysenur Akbulut, Henning Müller, Muhamed Barakovic, Lester Melie-Garcia, Meritxell Bach Cuadra, Cristina Granziera

**Affiliations:** a Translational Imaging in Neurology (ThINK) Basel, Department of Biomedical Engineering, Faculty of Medicine, University Hospital Basel and University of Basel, Basel, Switzerland; b Department of Neurology, University Hospital Basel, Basel, Switzerland; c Research Center for Clinical Neuroimmunology and Neuroscience Basel (RC2NB), University Hospital Basel and University of Basel, Basel, Switzerland; d MedGIFT, Institute of Informatics, School of Management, HES-SO Valais-Wallis University of Applied Sciences and Arts Western Switzerland, Sierre, Switzerland; e Clinical Trial Unit, Department of Clinical Research, University Hospital Basel, University of Basel, Basel, Switzerland; f CIBM Center for Biomedical Imaging, Lausanne, Switzerland; g Radiology Department, Lausanne University Hospital (CHUV) and University of Lausanne, Lausanne, Switzerland; h Ankara University School of Medicine, Ankara, Turkey; i Nuclear Medicine and Molecular Imaging Department, Lausanne University Hospital (CHUV) and University of Lausanne, Lausanne, Switzerland; j The Sense Research and Innovation Center, Lausanne and Sion, Switzerland

**Keywords:** MRI, Multiple sclerosis, Systematic review, Lesion segmentation, Lesion detection

## Abstract

Introduction: Over the past few years, the deep learning community has developed and validated a plethora of tools for lesion detection and segmentation in Multiple Sclerosis (MS). However, there is an important gap between validating models technically and clinically. To this end, a six-step framework necessary for the development, validation, and integration of quantitative tools in the clinic was recently proposed under the name of the Quantitative Neuroradiology Initiative (QNI).

Aims: Investigate to what extent automatic tools in MS fulfill the QNI framework necessary to integrate automated detection and segmentation into the clinical neuroradiology workflow.

Methods: Adopting the systematic Cochrane literature review methodology, we screened and summarised published scientific articles that perform automatic MS lesions detection and segmentation. We categorised the retrieved studies based on their degree of fulfillment of QNI’s six-steps, which include a tool’s technical assessment, clinical validation, and integration.

Results: We found 156 studies; 146/156 (94%) fullfilled the first QNI step, 155/156 (99%) the second, 8/156 (5%) the third, 3/156 (2%) the fourth, 5/156 (3%) the fifth and only one the sixth.

Conclusions: To date, little has been done to evaluate the clinical performance and the integration in the clinical workflow of available methods for MS lesion detection/segmentation. In addition, the socio-economic effects and the impact on patients’ management of such tools remain almost unexplored.

## Highlights

• There is lacking knowledge about how automated tools for lesion detection/segmentation in multiple sclerosis (MS) perform within a clinical setting and about how they might be integrated in clinical practice.
• The value and economic impact of those tools on patient management is unclear.
• The development of new tools for automated MS lesions detection/segmentation should include their integration in the clinical workflow.

## 1 Introduction

Multiple sclerosis (MS) is an inflammatory demyelinating disease of the central nervous system, which affects almost 3 million people worldwide (Walton et al., 2020). MS is the most prevalent neurological disease among young adults, and it is associated with a progressive increase in disability, which can significantly affect an individual’s quality of life as well as impose a substantial economic burden on patients, their families and the entire society (Feinstein, 2004). MS mostly exhibits focal inflammatory and degenerative lesions, but also diffused brain and spinal cord damage, which ultimately results in permanent brain volume loss (Reich et al., 2018). Hence, assessing the impact of neuroinflammation and neurodegeneration in patients through the identification of adequate imaging biomarkers is fundamental.

**Fig. 1:**
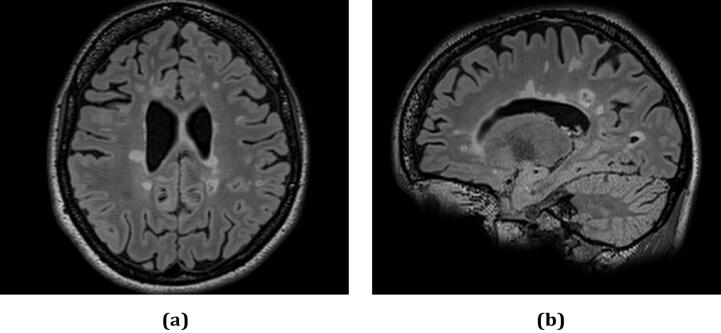
MS lesions in brain MRI axial (a) and sagittal (b) view. The figure comes from a dataset described in Lesjak et al. (2016).

MS diagnosis requires the demonstration of dissemination in space (i.e., specific regions of the brain and spinal cord must be affected by areas of focal inflammation/damage, which are named plaques or lesions) and time (i.e., assessment of the increase in lesions’ number and volume over time). The information provided by Magnetic Resonance Imaging (MRI) can address both requirements and, therefore, it is essential for MS diagnosis (Thompson et al., 2018). Fig. 1 shows the appearance of MS lesions in brain MRI.

The process of MS lesion detection and segmentation is usually performed manually by trained neuroradiologists and it is a time-consuming task and prone to errors (Egger et al., 2017). As a consequence, the development of automatic tools to support this procedure is urgently needed.

To date, several automated approaches have been proposed to support this key task, leading to a plethora of tools (reviewed in Llado et al., 2012; García-Lorenzo et al., 2012; Alrabai et al., 2022, Zeng et al., 2020, Ma et al., 2022, Diaz-Hurtado et al., 2022, Commowick et al., 2023) that are more or less mature towards clinical application and use. In the last 15 years, many international challenges, organised in the context of the Medical Image Computing and Computer Assisted Intervention (MICCAI) conference (Commowick et al., 2018, Kuijf et al., 2019, Styner et al., 2008, Commowick et al., 2021) and the International Symposium on Biomedical Imaging (ISBI) (Carass et al., 2017), provided benchmark datasets to promote a fair evaluation. In addition, the Shifts Challenge (Malinin et al., 2022) focused on the estimation of robustness and uncertainty of such methods.

To facilitate the adoption of automated image analysis tools in the practice of clinical neuroradiology, Goodkin et al. (2019) proposed a framework based on a sequence of six steps named Quantitative Neuroradiology Initiative (QNI). The six steps can be summarised in providing:

1. the target **clinical area** and **biomarkers**;
2. the structure of the **automated method**;
3. a **quantitative report**;
4. a **technical and clinical validation**;
5. details about the **integration** into the clinical workflow;
6. an **in-use evaluation**.

Although these requirements were originally applied to the radiological assessment of dementia, the framework was adopted later on to conduct systematic reviews on commercial volumetric MRI reporting tools in dementia (Pemberton et al., 2021) and MS (Mendelsohn et al., 2022). While in the present work we aim to present the state of the art of scientific literature, the mentioned reviews strictly focused on studies related to commercial devices.

Table 1 describes the requirements to fulfill the six QNI steps. The first and second steps include the identification of the target clinical area, the associated imaging biomarkers (lesional in the case of MS), the automated model’s structure, and reference datasets. A third phase consists of filing a visually informative quantitative report, to be integrated into the radiology report. The fourth step relates to the technical and pre-use clinical validation, which encapsulates a “credibility” and “accuracy” study. The former concept suggests a data quality check and a review of the technical performance of the method. The latter term refers to a blinded rating of a limited number of cases and an assessment of the clinical reporting process: radiologists’ accuracy and reporting efficiency should be examined, with and without the automated tool, in the closest possible environment to the usual radiology setting. The fifth step is the integration of tools into the clinical workflow, from data format compatibility to data protection and the joint visualisation of Digital Imaging and Communications in Medicine (DICOM) series and model output. The final phase describes an in-use pipeline evaluation for what concerns patient management and the socio-economic impact of the tool. Key concepts are the smoothness of the tool’s integration into a hospital’s radiology department, speed of diagnosis, cost in resources, productivity, general perception, and mid-term economic impact.

**Table 1:** Description of QNI steps.

	**QNI** step	**Requirements**
	**1**^**st**^	area of clinical need and corresponding imaging biomarkers (lesional for MS)
	**2**^**nd**^	structure of automatic method, benchmark dataset for training/testing
	**3**^**rd**^	quantitative (radiology) report with clinically and visually meaningful information
	**4**^**th**^	technical and clinical validation (quality check, with and without tool reporting assessment)
	**5**^**th**^	integration into the clinical workflow (data format compatibility and protection, input–output viewer)
	**6**^**th**^	in-use evaluation (patient management, socio-economic impact)

With these criteria, the proposed review analyses to what extent current literature of reporting automatic tools for detection and segmentation of MS lesions follows the QNI steps and, thus, considers the integration into the clinical routine.

## 2 Material and methods

In this review, we adopt the methodology described in the “Cochrane Handbook for Systematic Review of Interventions” (Lefebvre et al., 2022) to collect published articles till June 2023. To broaden and differentiate the screening pool, we targeted two databases, respectively medicine- and engineering-oriented: PubMed (https://pubmed.ncbi.nlm.nih.gov/) and IEEE (https://ieeexplore.ieee.org/Xplore/home.jsp). We adapted Cochrane’s threefold subdivision of screening keywords to our case, from “population, intervention and study design” to “population, task, and design of the tool”. While population refers to clinically confirmed MS patients, task and design describe what we expect as the automatic model’s first output and general characteristics.

By searching within both databases using Cochrane’s threefold subdivision of keywords, 770 studies were extracted (123 from IEEE and 647 from PubMed). Please note the word including a * is a “wild-card”, covering suffixes from a word stem, such as “automat*” stands for “automated” and “automatic”:

1. “multiple sclerosis”;
2. “segment*” OR “detect*”;
3. “machine learning” OR “deep learning” OR “automat*” OR “digital tool”.

The above criteria were applied to all metadata, including title, abstract, and keywords.

### 2.1 Study inclusion criteria

Screened articles were included in the review when they met all the following inclusion criteria:

1. original research published after 2011 in academic peer-reviewed journals or conferences in the English language;
2. studies targeting fully automatic detection or segmentation of white matter non-enhanced (without contrast agents) lesions, as either a primary or a secondary objective;
3. studies targeting brain MRI modalities;
4. studies targeting clinical MS population alone or mixed with patients with a clinically isolated syndrome (CIS, a first symptomatic episode of potential MS);
5. studies performing either technical, clinical, or in-use validation.

As a consequence, papers including a wider population than MS (in separate datasets), performing longitudinal or cross sectional evaluations, presenting a different primary goal or other lesion types (Rosa et al., 2022), have been reported in this review only if they also met the mentioned conditions. For each QNI framework’s step, the methodology of reviewed articles was discussed and evaluated as compliant or not compliant. It must be noted that failure to comply with some steps towards clinical use of those methods does not imply any superficiality in the methodology applied. It indicates, instead, that an article focuses primarily on other objectives.

In our work we distinguish among technical, clinical and in-use assessment as follows:

• **Technical** validation: comparing results to manual segmentation and/or state-of-the-art segmentation software, and data quality checks.
• **Clinical** validation: refers to any evaluation of the tool’s impact on clinical management, diagnostics, and reliability with respect to the reference annotated “ground truth”.
• **In-use** evaluation: includes any study measuring how easily the tool can be integrated into reporting workflow, benefits for patients, general perception, and socio-economic effects of the tool.

**Fig. 2:**
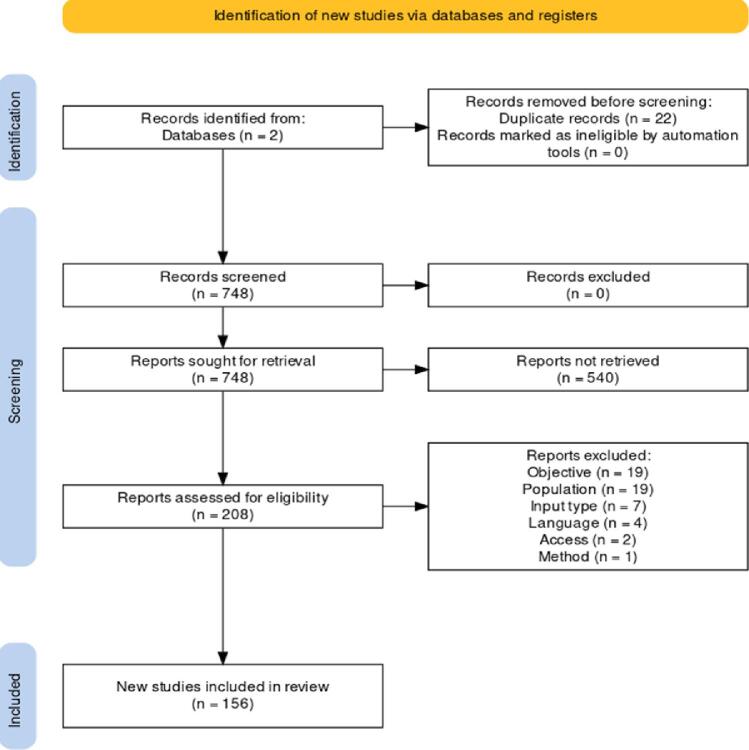
PRISMA flowchart applied during the screening process. The terms “objective”, “population”, “input type”, “language” refer to the inclusion criteria of Section 2.1. The term “access” refers to an exclusion due to the impossibility to access the full text of a paper. The term “method” refers to “not fully automatic methods”.

Merging results from the two databases, 22 records were excluded as duplicates leading to 748 studies to further review. Upon examination of the pool of abstracts, 562 occurrences were not retrieved as not compliant with the inclusion criteria. After carefully reviewing the full texts from the remaining 208 studies, 52 articles were further excluded due to their objective, population (e.g., dementia), input type (e.g., synthetic data), language, availability and method (only fully automatic methods were considered). The PRISMA flow diagram (Page et al., 2021) describing the procedure to select 156 studies to include in the review is reported in Fig. 2.

The search strategy was peer-reviewed by S.S., an experienced information specialist within our team. All data used in the review are available and can be accessed through PubMed and IEEE databases.

## 3 Results

Following the described methodology, 156 studies were identified (see the list of abbreviations in Table 2 and first columns of Table 3, Table 4, Table 5), which met all the inclusion criteria (Fig. 2).

**Table 2:** List of abbreviations.

**Abbreviation**	**Meaning**
FLAIR	fluid attenuated inversion recovery
MPRAGE	magnetisation-prepared rapid gradient echo
MP2RAGE	magnetisation-prepared 2 rapid gradient echo
PD-w	proton density weighted
T1-w	T1-weighted
T2-w	T2-weighted
DIR	double inversion recovery
PSIR	phase-sensitive inversion recovery
DP	diffusion perfusion
MNI	Montreal neurological institute
CLAHE	contrast limited adaptive histogram equalization
GM	gray matter
WM	white matter
CSF	cerebro spinal fluid
EPI	echo-planar imaging
CNN	convolutional neural network
FOV	field of view
SVM	support vector machine
FCNN	fully connected neural network
(A) VD	(absolute) volume difference
DSC	dice score
(L) TPR	(lesion) true positive rate
(L) FPR	(lesion) false positive rate
b.c.	bias field correction
reg.	registration
PPV	positive predicted value
TNR	true negative rate
FNR	false negative rate
IoU	intersection over union
AUC	area under the curve
NPV	negative predicted value
SI	similarity index
(p) AUC	partial area under the curve
HC	healthy controls
HD	Hausdorff distance
SD	surface distance

**Table 3:** Studies’ information containing details on datasets, inputs, and architecture of the automatic algorithm, pre-processing steps, and evaluation metrics (part 1).

	**Data**	**Inputs**	**Method**	**Pre-processing**	**Evaluation metrics**	**QNI steps fulfilled**	
(Yildirim and Dandil, 2021b)	MICCAI 2008, ISBI 2015	FLAIR	Mask R-CNN	/	DSC, AVD, lesion-wise TPR and FPR	1st, 2nd	
(Ghosal et al., 2020)	MICCAI 2016	T1-w, MPRAGE, FLAIR, T1-w gadolinium and T2/DP contrast enhanced	U-Net	denoising, intensity correction and skull-stripping	DSC accuracy, sensitivity, specificity	1st, 2nd	
(Kats et al., 2019)	ISBI 2015	T1-w, T2-w, PD-w and FLAIR	U-Net	/	DSC, precision, recall	1st, 2nd	
(Kumar et al., 2019)	MICCAI 2016	T1-w, MPRAGE, FLAIR, T1-w gadolinium-enhanced and T2/DP contrast enhanced	combination of Dense- and U-Net	remove null slices, z-score normalisation	DSC sensitivity, specificity, accuracy	1st, 2nd	
(Vang et al., 2020)	ISBI 2015	FLAIR	Mask R-CNN	skull stripping, b.c., z-score normalisation	DSC, precision, LTPR, LFPR, sensitivity	1st, 2nd	
(Joshi and Sharma, 2022)	MICCAI 2008 and 30 extra MS	T1-w, T2-w and FLAIR	CNN and graph convolutional networks	skull stripping, b.c.	DSC	1st, 2nd	
(Kolarik et al., 2021)	MICCAI 2016	FLAIR	VGG-16 encoder, residual U-Net decoder	normalisation	DSC and recall	1st, 2nd	
(Zhang et al., 2019)	ISBI 2015 and 15 extra simulated MS	T1-w MPRAGE, FLAIR	2D fully convolutional densely connected network	ISBI: b.c., skull and dura stripping, 2nd b.c. and MNI reg.; extra: reg. to T1-w, skull stripping, b.c.	DSC, precision, recall, LFPR, LTPR, VD	1st, 2nd	
(Kamraoui et al., 2021)	ISBI 2015, MICCAI 2016, extra 43 subjects	T1-w and FLAIR	U-Net	ISBI: b.c., skull and dura stripping, b.c., MNI reg; MICCAI: denoising, reg. on FLAIR, skull stripping, b.c., MNI reg.; extra: denoising, MNI reg., skull stripping, b.c., denoising, normalisation	DSC, precision, recall, LTPR, LFPR	1st, 2nd	
(Chen et al., 2021)	ISBI 2015, extra 157 MS	FLAIR	local attention feature and graph attention clustering	ISBI: skull stripping, reg. and normalisation; extra: b.c., normalisation, resampling	DSC, TPR, LTPR, LFPR, absolute VD	1st, 2nd	
(Billot et al., 2021)	MICCAI 2016, ISBI 2015	T1-w and FLAIR	U-Net	ISBI: skull stripping	DSC and brain ROI	1st, 2nd	
(Alijamaat et al., 2021)	MICCAI 2016	MPRAGE, FLAIR, T1w gadolinium, PD-w	CNN with wavelet pooling	remove null slices, 0–1 normalisation	accuracy, TPR, DSC	1st, 2nd	
(Basaran et al., 2022)	MSSEG-2	FLAIR	nnU-Net	skull stripping, b.c., baseline and follow-up reg.	F1, DSC, volume of FP	1st, 2nd	
(Hashemi et al., 2018)	MICCAI 2016, ISBI 2015	MPRAGE, FLAIR, T1 contrast enhanced, PD-w, T2; MPRAGE, FLAIR, PD-w, T2	U-Net, FC Dense-Net	reg.	DSC, recall, F2, Jaccard index, LTPR, LFPR, VD	1st, 2nd	
(Roura et al., 2015)	MICCAI 2008, extra 14 MS	T1-w and FLAIR	Statistical Parametric Mapping	skull stripping, denoising, b.c., MNI reg.	DSC, TPR, PPV	1st, 2nd	
(Essa et al., 2020)	MICCAI 2008	T2-w and FLAIR	R-CNN	MNI reg., skull stripping, b.c.	TPR, PPV, DSC, VD	1st, 2nd	
(Zhang et al., 2021)	ISBI 2015, extra 176 MS	T1-w, FLAIR, T2-w, anatomical coordinates	encoder-decoder backbone, anatomical convolutional modules, region-based loss modules	MNI reg.	DSC, precision, sensitivity, F1, LTPR, LFPR, Lesion F1	1st, 2nd	
(Sadeghibakhi et al., 2022)	ISBI 2015	T1-w and FLAIR	ResNet	skull stripping, normalisation, MNI reg., CLAHE	DSC, LTPR, LFPR, absolute VD	1st, 2nd	
(Brosch et al., 2016)	MICCAI 2008, ISBI 2015, extra 195 subjects	T1-w, T2-w, PD-w, FLAIR or T1-w, T2-w, PD-w	CNN with shortcuts	skull stripping, 0–1 normalisation, reg.	DSC, VD, LTPR, LFPR	1st, 2nd	
(Ackaouy et al., 2020)	MICCAI 2016	T1-w and FLAIR	CNN	denoising, skull stripping, b.c.	DSC and F1	1st, 2nd	
(Ashtari et al., 2022)	MSSEG-2	FLAIR	U-Net	zero regions removal, z-score normalisation, resampling	DSC, HD, sensitivity, PPV, F1, number and volume of predicted lesions	1st, 2nd	
(Fenneteau et al., 2021)	ISBI 2015	FLAIR	U-Net	z-score normalisation	DSC, sensitivity, precision	1st, 2nd	
(Sarica and Seker, 2022)	MSSEG-2	FLAIR	U-Net	skull stripping, b.c., normalisation, baseline and follow-up fusion	DSC, F1, n. and volume of predictions, PPV, sensitivity, specificity, mean SD	1st, 2nd	
(Aslani et al., 2019)	ISBI 2015, extra 37 MS	T1-w, T2-w, FLAIR	ResNet	skull stripping, MNI reg., normalisation	DSC, LTPR, LFPR, avg symmetric SD, HD, PPV, VD	1st, 2nd	
(Valverde et al., 2017)	MICCAI 2008	T1-w, T2-w, FLAIR	CNN	co-reg.	VD, TPR, FPR	1st, 2nd	
(Filho, 2017)	MICCAI 2016, extra 17 subjects	T1-w and FLAIR	iterative contrast enhancement and logistic classification	MNI reg., WM-GM segmentation, b.c., denoising	sensitivity, specificity, DSC, volume similarity	1st, 2nd	
(Guizard et al., 2015)	MICCAI 2008, extra 108 MS	T1-w, T2-w, FLAIR	rotation-invariant multi-contrast non-local mean segmentation	denoising, normalisation, MNI reg., skull stripping	DSC, TPR LTPR, PPV, LPPV, VD, FPR, symmetric SD	1st, 2nd	
(Abdullah et al., 2012)	MICCAI 2008, extra 10 MS and synthetic data	T1-w, T2-w and FLAIR	SVM with textural features, position features, co-registered intensities, tissues priors and neighbouring blocks features	MNI reg., normalisation	DSC, detected lesion load, TPR, PPV	1st, 2nd	
(Geremia et al., 2011)	MICCAI 2008	T1-w, T2-w and FLAIR	discriminative random decision forest	sub-sampling and cropping, b.c., normalisation, MNI reg., segmentation of WM, GM, CSF	TNR, TPR, FPR, PPV, volume overlap, VD, symmetric SD	1st, 2nd	
(Joshi and Sharma, 2021)	50 MS	T1-w, T2-w and FLAIR	graph convolutional network and cnn autoencoder	b.c.	DSC, precision and loss	1st, 2nd	
(Sepahvand et al., 2020)	multi-centric 886 MS	T1-w, T2-w, PD-w and FLAIR	U-Net	skull stripping, b.c., normalisation, MNI reg.	AUC, specificity and sensitivity	1st, 2nd	
(Papadopoulos et al., 2022)	30 MS	FLAIR	U-Net	cropping window on label mask, 0–1 normalisation	accuracy, IoU, DSC, precision, recall	1st, 2nd	
(Nair et al., 2019)	multi-centric 1064 MS	T2-w, T1-w, PD-w, FLAIR	CNN	skull stripping, b.c., MNI reg.	ROC with TPR/FPR at voxel and lesion level	1st, 2nd	
(Rosa et al., 2020)	54 MS	MP2RAGE and FLAIR	U-Net	co-reg.	DSC, absolute VD, TPR, LTPR, FPR, LFPR, WML and CL detection rate	1st, 2nd	
(Gabr et al., 2019)	multi-centric 1008 MS	T1-w, T2-w, PD-w and FLAIR	U-Net	denoising, skull stripping, b.c., normalisation	DSC, TPR, FPR, classification based on volume	1st, 2nd	
(Hermann et al., 2021)	bi-centric 35 MS	MR fingerprinting EPI	U-Net	denoising, distortion correction	DSC and lesion detection rate	1st, 2nd	
(Rakic et al., 2021)	multi-centric 159 MS	T1-w and FLAIR	U-Net with attention gate layers	MNI reg., skull stripping, z-score normalisation	lesion/voxel-wise DSC, confusion matrix, lesion load	1st, 2nd	
(Krüger et al., 2021)	multi-centric 1809 MS	T1-w and FLAIR	CNN	strong artifacts exclusion, reg. on FLAIR	detection (1 voxel overlap), sensitivity, F1 lesion-wise, DSC voxel-wise	1st, 2nd	
(Krishnan et al., 2021)	multi-centric 1574 MS	T1-w and FLAIR or T1-w post-contrast and FLAIR	2.5D U-Net	b.c., MNI reg., skull stripping	PPV, sensitivity, absolute VD	1st, 2nd	
(Elliott et al., 2013)	multi-centric 255 MS	T1-w, T2-w and FLAIR	Bayesian and random-forest based lesion-level classifier	skull stripping, b.c., normalisation	sensitivity, false detection rate	1st, 2nd	
(Cabezas et al., 2014)	multi-centric 45 MS	T1-w, PD-w, T2-w, FLAIR, prob. maps of CSF, GM, WM, an outlier map, 800 region-based comparison meta-features	Gentleboost classifier	skull stripping, b.c., denoising, normalisation, reg.	DSC	1st, 2nd	
(Sajja et al., 2006)	23 MS	T2-w and FLAIR	Parzen estimator with Gaussian kernel	removal of lesions close to the brain surface, FP/FN minimisation	similarity index, % of correct, over- and under-estimation	1st, 2nd	
(Sweeney et al., 2013)	bi-centric 208 MS	T1-w, T2-w, PD-w and FLAIR	logistic regression with gaussian kernel	MNI reg., b.c., skull stripping, intensity thresholding, normalisation, multi-resolution smoothed volumes	FPR, sensitivity and DSC	1st, 2nd	
(Steenwijk et al., 2013)	20 MS	T1-w and FLAIR	k-nearest neighbour	skull stripping, MNI reg., b.c.	DSC, sensitivity	1st, 2nd	
(Schmidt et al., 2011)	52 MS	T1-w and FLAIR	adaptive maximum a posteriori estimations and Markov random field	b.c., MNI reg., classification of CSF, WM, GM	DSC, correlation and regression of lesion volume	1st, 2nd	
(Todea et al., 2023)	multi-centric 206 MS	MPRAGE and FLAIR at 2 timepoints	k-nearest neighbour	b.c., normalisation, reg.	sensitivity, specificity, accuracy, F1, PPV, NPV	1st, 2nd	
(Gessert et al., 2020)	bi-centric 122 MS	FLAIR	LST toolbox vs U-Net and two path CNNs with attention-guided interaction modules	z-score normalisation	LTPR, LFPR, DSC	1st, 2nd	
(Hitziger et al., 2022)	MSSEG-2	FLAIR	U-Net	MNI reg., crop FOV to area around brain, z-score normalisation	F1, recall, precision, DSC	1st, 2nd	
(Mengin et al., 2022)	MSSEG-2, extra 17 MS	FLAIR	U-Net	z-score normalisation	DSC, sensitivity, PPV, F1	1st, 2nd	
(Krüger et al., 2020)	multi-centric 1791 MS	FLAIR	U-Net	/	LTPR, LFPR, DSC lesion-wise	1st, 2nd

**Table 4:** Studies’ information containing details on datasets, inputs, and architecture of the automatic algorithm, pre-processing steps, and evaluation metrics (part 2).

	**Data**	**Inputs**	**Method**	**Pre-processing**	**Evaluation metrics**	**QNI steps fulfilled**	
(Schmidt et al., 2019)	bi-centric 60 MS	FLAIR	analysis of FLAIR intensities distribution	tissue classification, b.c. and co-reg.	DSC, FPR, TPR	1st, 2nd	
(Combès et al., 2021)	multi-centric 54 MS	T1-w, T2-w, FLAIR	FCNN	orientation in RAS coordinates, skull stripping, baseline and follow-up reg., cropping, b.c., intensity histogram linear rescaling and Nyul standardisation	n. of lesions and annotation and reporting time with and without tool, inter-rater variability	1st, 2nd, 3rd, 4th	
(Jannat et al., 2021)	30 MS and 100 controls	T1-w, T2-w and FLAIR	CNN	/	precision, recall, F1	1st, 2nd	
(Salem et al., 2020)	60 MS	T1-w, T2-w, PD-w and FLAIR	FCNN	skull stripping, b.c., Nyul standardisation	TPF, FPF, DSC	1st, 2nd	
(Zhang et al., 2022)	multi-centric 507 MS	FLAIR	2D U-Net	normalisation and resampling	DSC, sensitivity, precision	1st, 2nd	
(Rachmadi et al., 2019)	bi-centric 40 MS	FLAIR	irregularity maps generation	brain and CSF mask extraction, co-reg. and b.c.	DSC, PPV, spec, TPR, non-parametric Spearman’s correlation coefficient	1st, 2nd	
(Chen et al., 2021)	ISBI 2015	FLAIR, MPRAGE, T2-w, PD-w	U-Net	b.c., z-score normalisation	DSC, PPV, TPR, LFPR, LTPR	1st, 2nd	
(Mehta et al., 2021)	multi-centric 1073 MS	T1-w, T2-w, PD-w and FLAIR	Bayesian U-Net and another U-Net		AUC with TPR and FDR	1st, 2nd	
(Opbroek et al., 2014)	multi-centric 70 MS	T1-w, T2-w and FLAIR	reduced SVM on 33 features	Nyul standardisation	relative AVD, average symmetric SD, TPR, FPR	1st, 2nd	
(de Oliveira et al., 2022)	ISBI 2015, MICCAI 2016	FLAIR	U-Net	anisotropic diffusion filter, normalisation, skull stripping, b.c.	DSC, accuracy, precision, sensitivity, specificity	1st, 2nd	
(Yamamoto et al., 2022)	28 MS	FLAIR	CNN	denoising	absolute VD, PPV, TPR, DSC, HD, F1	1st, 2nd	
(McKinley et al., 2019)	multi-centric 139 MS	T1-w, T2-w and FLAIR	FCNN	skull stripping, co-reg.	DSC	1st, 2nd	
(Zhang et al., 2021b)	multi-centric 200 MS	T1-w, T2-w and FLAIR	U-Net	normalisation	DSC, LTPR, LPPV, lesion-wise F1	1st, 2nd	
(Abolvardi et al., 2019)	ISBI 2015	FLAIR	U-Net	reg.	DSC	1st, 2nd	
(Fenneteau et al., 2021a)	ISBI 2015, extra 30 MS	FLAIR	MPU-net	skull stripping, z-score normalisation	DSC	1st, 2nd	
(de Oliveira et al., 2020)	ISBI 2015, extra 5 MS	T1-w and FLAIR	CNN	skull stripping, b.c.	volume of lesions	1st, 2nd	
(Yildirim and Dandil, 2021a)	38 MS	T2-w	mask R-CNN with ResNet101 as backbone	/	DSC, volume overlap error, LTPR, LFPR	1st, 2nd	
(Tran et al., 2022)	30 MS	T1-w and FLAIR	intensity-based	b.c.	WM hyperintensities volume agreement, DSC, FPR, TPR, F1 score	1st, 2nd, 3rd	
(Cavedo et al., 2022)	130 images multi-centric, different populations	FLAIR	intensity-based	/	WM hyperintensities volume, DSC, relative VD, absolute volume error	1st, 2nd, 3rd	
(Brune et al., 2020)	56 MS	MPRAGE and FLAIR	intensity-based	/	lesion count of tool vs neuroradiologists, single and multiple timepoints	1st, 2nd, 3rd	
(Jain et al., 2017)	22 MS	T1-w and FLAIR	maximum a posteriori model on image intensities of both time points	b.c., normalisation	DSC, F1, LTPR, LFPR, AVD	1st, 2nd	
(Van Hecke et al., 2021)	batches of 10 and 25 MS, plus 87 subjects with CIS and MS	T1-w and FLAIR	U-Net with attention gate layers	MNI reg., skull stripping, z-score normalisation	with vs without tool performances, surveys on patient’s perspective	1st, 2nd, 3rd, 4th, 5th, 6th	
(Sousa et al., 2021)	ISBI 2015, MICCAI 2016, 33 MS bicentric	T1-w, FLAIR	CNN	skull stripping, b.c.	DSC, PPV, AVD	1st, 2nd	
(Elsebely et al., 2021)	MICCAI 2008	FLAIR	ensemble of SVMs and decision tree	contrast-brightness correction	DSC, accuracy, n. of TP/FP/FN, sensitivity, PPV	1st, 2nd	
(Bhanumurthy and Anne, 2016)	MICCAI 2016	FLAIR	modified histon based fast fuzzy C-means	normalisation	FPR, FNR, specificity, sensitivity, accuracy	1st, 2nd	
(Ghodhbani et al., 2022)	MICCAI 2008, ISBI 2015	T1-w, T2-w and FLAIR	U-Net	/	DSC	1st, 2nd	
(Roy et al., 2013)	MICCAI 2008	T1-w, T2-w and FLAIR	SVM	MNI reg., skull stripping, z-score normalisation		1st, 2nd	
(Chen et al., 2020)	MICCAI 2016, ISBI 2015	PD-w, T1-w, T2-w and FLAIR	network with attention and graph convolution features	/	DSC, PPV, LFPR, LTPR, VD	1st, 2nd	
(Tomas-Fernandez and Warfield, 2015)	synthetic and MICCAI 2008	FLAIR	intensity-based using reference healthy population	b.c., denoising	PPV, LTPR, LFPR	1st, 2nd	
(Ghribi et al., 2017)	MICCAI 2008, ISBI 2015 and 70 MS and HC	FLAIR	gaussian mixture model	b.c., skull stripping, denoising, z-score normalisation	DSC, PPV, LTPR, LFPR, SD, VD	1st, 2nd	
(Salem et al., 2019)	65 MS/CIS, synthetic and ISBI 2015	T1-w and FLAIR	FCNN	b.c.	mean square error, structural similarity index, DSC, sensitivity, precision	1st, 2nd	
(Hashemi et al., 2018)	MICCAI 2016, ISBI 2015	MPRAGE, PD-w, FLAIR, T2-w	U-Net vs FC DenseNet	/	DSC, F2, sensitivity, precision, Jaccard index, PPV, LTPR, LFPR, VD	1st, 2nd	
(Hou et al., 2019)	ISBI 2015	FLAIR, t1-w, T2-w, PD-w	cross attention densely-connected network	/	DSC, Jaccard index, PPV, TPR, LFPR, LTPR, VD, SD	1st, 2nd	
(Rondinella et al., 2023)	ISBI 2015	FLAIR	U-Net with attention mechanism	skull stripping, normalisation and black images removal	DSC, sensitivity, specificity, extra fraction, IoU, PPV, NPV	1st, 2nd	
(Weiss et al., 2013)	MICCAI 2008	FLAIR	dictionary learning with sparsity constraint	skull stripping, normalisation	DSC, TPR, PPV	1st, 2nd	
(Homayoun and Ebrahimpour-Komleh, 2017)	MICCAI 2008	FLAIR	artificial neural network	skull stripping, b.c., denoising, normalisation	sensitivity, specificity, FPR, FNR, SI	1st, 2nd	
(Andresen et al., 2022)	MS-SEG2	FLAIR	CNN	skull stripping, 0–1 normalisation	F1, sensitivity, PPV (detection); DSC, SD, HD (segmentation)	1st, 2nd	
(Valencia et al., 2022)	MS-SEG2 and 136 CIS	FLAIR and synthetic T1-w	FCNN	MNI reg., skull stripping, 0–1 normalisation	sensitivity, FDR, precision	1st, 2nd	
(Salem et al., 2022)	MS-SEG2	FLAIR	U-Net	skull stripping, Nyul normalisation, b.c.	F1, PPV, sensitivity, DSC	1st, 2nd	
(Freire and Ferrari, 2016)	ISBI 2015	FLAIR	intensity-based	denoising, b.c.	DSC, TPR, FPR, AVD	1st, 2nd	
(Sarica et al., 2022)	ISBI 2015, MICCAI 2016	T1-w, T2-w and FLAIR	residual U-Net	skull stripping, normalisation and zero padding (ISBI), denoising, skull stripping, b.c., normalisation (MICCAI)	DSC, PPV, LTPR, LFPR, AVD	1st, 2nd	
(Shahab et al., 2021)	ISBI 2015	T1-w, T2-w and FLAIR	CNN	denoising, b.c., skull stripping, normalisation	DSC, Jaccard index, PPV, TPR, LFPR, LTPR, AVD	1st, 2nd	
(Jog et al., 2015)	MICCAI 2008 and in-house 49 MS	T1-w, FLAIR, T2-w (MPRAGE for in-house)	decision trees	MNI reg., skull stripping, normalisation, b.c.	TPR, PPV, lesion volume	1st, 2nd	
(Knight et al., 2018)	96 MS multicentric, MICCAI 2016, ISBI 2015	FLAIR	voxel-wise logistic regression	MNI reg., b.c., normalisation	SI, precision and recall	1st, 2nd	
(Valcarcel et al., 2018)	98 MS and ISBI 2015	combinations of MPRAGE, PD-w, FLAIR, T2-w	local-level logistic regression	MNI reg., b.c., skull stripping, z-score normalisation	DSC, pAUC	1st, 2nd	
(Gao et al., 2014)	MICCAI 2008	T1-w, T2-w and FLAIR	energy minimisation and non-local means algorithm	b.c., reg.	DSC, specificity, FNR, VD	1st, 2nd	
(Krishnan et al., 2023)	ISBI 2015, MICCAI 2016 and multicentric double blinded trial 798 + 714 + 416 MS	T1-w, T2-w and FLAIR	U-Net	MNI reg., b.c., skull stripping	PPV, TPR, DSC on lesion volume (segmentation), LPPV, LTPR, LFPR on lesion count (detection), also AVD on ISBI		
(Mechrez et al., 2016)	MICCAI 2008 and 38 MS	T1-w and FLAIR	intensity-based	b.c., skull stripping	VD, SSD, TPR, FPR	1st, 2nd	
(Bouzidi et al., 2020)	30 MS	T1-w, T2-w and FLAIR	otsu threshold and connected components filters	b.c., denoising	DSC, sensitivity, precision	1st, 2nd	
(Bijar et al., 2012)	20 MS	FLAIR	genetic algorithm and localised weighted filters	/	similarity criteria, overlap fraction, extra fraction	1st, 2nd	
(Narayana et al., 2018)	multi-center, double-blinded, and randomized phase III clinical trial 1008 MS	T1-w, T2-w, PD-w and FLAIR	CNN	skull stripping, b.c., normalisation	DSC	1st, 2nd	
(Tripoliti et al., 2019)	/		/	/	/	3rd, 5th	
(Abhale et al., 2022)	1000 MS from phase 3 multicentric trial	T2-w	FCNN	/	DSC	1st, 2nd	
(Zangeneh and Yazdi, 2016)	20 MS	FLAIR	gaussian mixture model and genetic algorithm	skull stripping	accuracy, number of FP, TP, FN, TN	1st, 2nd

**Table 5:** Studies’ information containing details on datasets, inputs, and architecture of the automatic algorithm, pre-processing steps, and evaluation metrics (part 3).

	**Data**	**Inputs**	**Method**	**Pre-processing**	**Evaluation metrics**	**QNI steps fulfilled**	
(Karpate et al., 2015)	16 MS and 20 HC	MPRAGE, T2-w, FLAIR	Least squares probabilistic classification	b.c., denoising	precision, recall	1st, 2nd	
(Mei et al., 2017)	10 MS	FLAIR, T1-w (also with gadolinium)	self-organising maps (nerual network)	/	topographic and quantisation errors	1st, 2nd	
(Zhang et al., 2018)	69 MS	T1-w and FLAIR	generative adversarial network	b.c. on T1-w	DSC, recall, precision, F1	1st, 2nd	
(Dachraoui et al., 2020)	30 MS	T1-w (also gadolinium), T2-w and FLAIR	Fuzzy C-Means clustering and geodesic models	skull stripping, denoising, contrast adjustment	number of TP, TN and precision	1st, 2nd	
(Deshpande et al., 2015)	14 MS	MPRAGE, PD-w, FLAIR	adaptive dictionary learning	b.c., skull stripping	PPV, sensitivity	1st, 2nd	
(Harmouche et al., 2014)	100 MS multicentric	T1-w, T2-w, PD-w, FLAIR (for half datasets)	Markov random fields	b.c., skull stripping, normalisation	DSC, sensitivity, PPR (ratio)	1st, 2nd	
(Nass et al., 2022)	30 MS	T1-w (also contrast enhanced), T2-w, FLAIR	Fuzzy C-Means	contrast adjustment	DSC	1st, 2nd	
(Zhang et al., 2022)	135 MS	FLAIR	2D U-Net	normalisation	DSC, sensitivity, precision	1st, 2nd	
(Ye et al., 2020)	38 MS	diffusion basis spectrum imaging, T1-w and T2-w	FCNN	normalisation	number of predictions, AUC, sensitivity, specificity, F1	1st, 2nd	
(Thakur et al., 2022)	200 MS per month for 10yrs	FLAIR	intensity subtraction between timepoints	b.c., skull stripping, resampling and reg.	number of clinical cases assessed with CAD, time per patient	1st, 2nd, 3rd, 5th	
(Fartaria et al., 2019)	25 MS bicentric	7T MP2RAGE	partial volume estimation and topological constraints	skull stripping	% of detected lesions, FPR, AVD, F1	1st, 2nd	
(Hosseinipanah et al., 2019)	>80 MS	FLAIR	ensemble of SVMs	normalisation	DSC, JI, sensitivity, specificity, PPV	1st, 2nd	
(Meier et al., 2017)	29 MS + 13 MS and 15 HC	T1-w, T2-w and FLAIR	intensity-based with 2 thresholds for supra- and infra-tentorial	b.c., skull stripping, tissue segmentation, normalisation	sensitivity, specificity, DSC, Jaccard index, PPV, HD, TPR	1st, 2nd	
(Jain et al., 2015)	30 MS	T1-w and FLAIR	intensity-based	skull stripping on T1-w	DSC, AVD, total lesion VD, precision, sensitivity	1st, 2nd	
(Bonanno et al., 2021)	20 MS	FLAIR	Watershed-Clustering algorithm	denoising	accuracy, sesnitivity, specificity, AUC	1st, 2nd	
(Huang et al., 2022)	20 MS	FLAIR	V-Net	b.c., skull stripping, tissue segmentation	DSC, HD, AVD, TPR, F1	1st, 2nd	
(Narayana et al., 2019)	multicentric, double blinded, randomized trial 1008 MS	T1-w, T2-w, PD-w and FLAIR in combinations	U-Net	skull stripping, b.c., normalisation, denoising	DSC, FPR, TPR	1st, 2nd	
(Arnold et al., 2022)	33 MS bicentric	T1-w, T2-w, FLAIR	local-level logistic regression	b.c., normalisation	DSC, TPR, FDR	1st, 2nd	
(Krüger et al., 2021)	1809 MS multicentric	FLAIR or T1-w and FLAIR	CNN	strong artifacts removal	sensitivity, PPV, F1, DSC	1st, 2nd	
(Fartaria et al., 2018)	39 MS	FLAIR and MPRAGE	outlier rejection and region growing vs fuzzy clustering	skull stripping, b.c.	detection rate, FPR, DSC, lesion volume	1st, 2nd	
(Karimian and Jafari, 2015)	25 MS bicentric	T1-w, T2-w, FLAIR	Gaussian mixture model	skull stripping, normalisation	DSC, accuracy, specificity, sensitivity	1st, 2nd	
(Sweeney et al., 2014)	98 MS	T1-w, T2-w, FLAIR	intensity-based	MNI reg., skull stripping, b.c., normalisation	DSC, AUC, computational time	1st, 2nd	
(Fartaria et al., 2015)	39 MS	FLAIR, DIR, MPRAGE, MP2RAGE	k-nearest neighbour	reg. to MP2RAGE, skull stripping, b.c., normalisation	detection rate, sensitivity, specificity, accuracy, DSC	1st, 2nd	
(Rosa et al., 2021)	44 MS and 12 HC	MPRAGE to generate synthetic MP2RAGE	generative adversarial network	skull stripping, z-score normalisation	detection rate (WML and cortical), DSC, AVD	1st, 2nd	
(Rovira et al., 2021)	100 MS	PD-w, T2-w, MPRAGE, FLAIR	CNN	skull stripping, b.c., normalisation	number of new/enlarging lesions, per patient mean new/enlarging lesions	1st, 2nd	
(Egger et al., 2016)	50 MS	FLAIR	intensity-based	/	DSC, AVD, lesion count	1st, 2nd	
(Cabezas et al., 2014)	45 MS	FLAIR	intensity-based	atlas reg., skull stripping, b.c., denoising	TPF, FPF, DSC at voxel and lesion level	1st, 2nd	
(Salem et al., 2017)	60 MS/CIS	T1-w, T2-w, PD-w, FLAIR	image subtraction and logistic regression	b.c., skull stripping, Nyul normalisation	TPF, FPF, DSC	1st, 2nd	
(Khotanlou and Afrasiabi, 2012)	15 MS	T1-w, T2-w and FLAIR	SVM	denoising, morphological operations to exclude non-brain area	SI, overlap fraction, extra-fraction	1st, 2nd	
(Valcarcel et al., 2018a)	40 MS	T1-w, T2-w, FLAIR	local level logistic regression	skull stripping, b.c., normalisation	DSC, pAUC, root mean square, detection and outline errors	1st, 2nd	
(Valcarcel et al., 2020)	94 MS and 40 MS	T1-w, T2-w, FLAIR	intensity-based	MNI reg., skull stripping, normalisation	lesion volume bias, absolute volume error	1st, 2nd	
(Dwyer et al., 2019)	multicentric 100 subjects, 192 MS/CIS, 15 MS, 125 MS and 76 HC	FLAIR	random forest classifier	b.c., normalisation	agreement with conventional T2-w lesion volume	1st, 2nd	
(Battaglini et al., 2014)	multicentric, randomized, double-blind, placebo-controlled Phase II clinical trial 103 MS (randomly select 19 MS)	T1-w, T2-w, PD-w	subtraction images between timepoints	skull stripping, normalisation	SI, lesion count	1st, 2nd	
(Le et al., 2019)	47 MS multicentric	FLAIR and T2-w combination (also T1-w depending on algorithm)	comparison of three algorithms	b.c., skull stripping	lesion VD, DSC, sensitivity, symmetric SD	1st, 2nd	
(Zhong et al., 2014)	26 MS	FLAIR	high spatial frequency suppression	b.c., skull stripping, CSF sulcus and ventricle segmentation	SI, lesion volume	1st, 2nd	
(Galimzianova et al., 2017)	30 MS	FLAIR	Markov random fields	skull stripping, b.c.	DSC, total lesion load	1st, 2nd	
(Schläger et al., 2022)	74 MS multicentric	T1-w and FLAIR to generate synthetic DIR	generative adversarial network	b.c., normalisation	new lesions count (location based), disease activity assessment	1st, 2nd	
(Subbanna et al., 2015)	1195 MS	T1-w, T2-w, PD-w, FLAIR	two levels of Markov Random Fields	b.c., skull stripping, normalisation	sensitivity, PPV	1st, 2nd	
(Galimzianova et al., 2015)	30 MS	T1-w, T2-w and FLAIR	stratified mixture models	b.c.	DSC, Jeffrey’s divergence	1st, 2nd	
(Spies et al., 2013)	10 MS	MPRAGE	tissue segmentation, stereotactic normalisation and voxelwise stat analysis	/	DSC	1st, 2nd	
(Ganiler et al., 2014)	20 MS	T2-w, PD-w combinations	image subtraction	b.c., skull stripping, normalisation, WM masking	sensitivity, FDR, DSC	1st, 2nd	
(Nguyen et al., 2018)	30 MS	FLAIR	subtraction image	b.c., skull stripping, normalisation	sesnitivity, specificity, human review time	1st, 2nd	
(Ong et al., 2012)	MICCAI 2008, 38 MS	T1-w, FLAIR	intensity-based	skull stripping, b.c.	lesion load, SI, Jaccard index, FPF, TPF	1st, 2nd	
(Cerasa et al., 2011)	11 MS	FLAIR	cellular neural network	skull stripping	DSC, total lesion load	1st, 2nd	
(Kuwazuru et al., 2011)	3 MS	T1-w, T2-w, FLAIR	SVM and artificial neural network	enhancement by subtraction of background	accuracy, SI, sensitivity, number of FP	1st, 2nd	
(Bilello et al., 2013)	88 MS	FLAIR	image subtraction	skull stripping, b.c.	with and without CAD: lesion count and location, PPV, NPV, sensitivity, specificity, efficiency, AUC, lesion-wise sensitivity, FPR and PPV, time spent, clinical reporting	1st, 2nd, 3rd, 4th, 5th	
(Hindsholm et al., 2021)	93 MS	FLAIR	2D CNN	normalisation, cropping or zero padding	DSC, recall, F1, precision, qualitative assessment of output lesion masks	1st, 2nd	
(Roy et al., 2015)	10 MS	MPRAGE and FLAIR	patch based with temporal information from timepoints	normalisation	DSC, LTPR, LFPR, AVD	1st, 2nd	
(Bouman et al., 2023)	198 MS	DIR or PSIR generated artificially from T1-w, T2-w, PD-w or FLAIR	U-Net-like	MNI reg., skull stripping, b.c.	lesions count, precision	1st, 2nd	
(Sitter et al., 2017)	69 MS + 1 CIS	2D FLAIR and 3D T1-w	multiple models comparison	MNI reg., b.c.	SI, volumes of FP and FN	1st, 2nd	
(Cabezas et al., 2016)	36 MS/CIS	FLAIR	image subtraction	skull stripping, b.c., normalisation	DSC, FPR, TPR	1st, 2nd	

### 3.1 Target population

In ten articles, MS patients were mixed with subjects presenting CIS (Salem et al., 2020, Jannat et al., 2021, Salem et al., 2019, Valencia et al., 2022, Salem et al., 2017, Dwyer et al., 2019, Sitter et al., 2017, Cabezas et al., 2016), or neuromyelitis optica spectrum disorders and cerebral small vessel disease (Zhang et al., 2022), or mild cognitive impairment, Alzheimer’s disease, Parkinson’s disease and frontotemporal dementia (Cavedo et al., 2022). The remaining studies targeted at least one dataset with only MS patients (see second columns of Table 3, Table 4, Table 5).

### 3.2 Magnetic resonance imaging

Fluid attenuated inversion recovery (FLAIR) was the most common MRI contrast used as input for the proposed automatic methods. It was used alone or in combination with a T1-weighted (T1-w) image, a T2-weighted (T2-w), a proton density weighted (PD-w) image, or with contrast enhancement (see third columns of Table 3, Table 4, Table 5). In six cases, the only input provided to the network were either T2-w images (Abhale et al., 2022, Yildirim and Dandil, 2021a), MPRAGE (Magnetisation-prepared rapid gradient echo) (Galimzianova et al., 2015, Spies et al., 2013), MP2RAGE (Magnetisation-prepared 2 rapid gradient echo) (Fartaria et al., 2019) or MR fingerprinting EPI (Echo-planar imaging) (Hermann et al., 2021). Less common contrasts, such as diffusion basis spectrum imaging (Ye et al., 2020), DIR (Fartaria et al., 2015, Schläger et al., 2022, Bouman et al., 2023) and PSIR (Bouman et al., 2023) were also adopted.

### 3.3 Datasets

The methods developed by 92 studies were (at least partially) based on datasets from international challenges: MICCAI 2008 (Styner et al., 2008), MICCAI 2016 (MS-SEG) (Commowick et al., 2018), MS-SEG2 (Commowick et al., 2021) and ISBI 2015 (Carass et al., 2017). Earlier works focused on relatively small cohorts due to the limited sample size provided in the challenges, such as 5 and 20 patients, respectively, in the training set of ISBI 2015 in Vang et al. (2020) and of MICCAI 2008 in Joshi and Sharma (2022).

A single case (Tripoliti et al., 2019) did not provide any reference dataset. The authors proposed the architecture of a tool for the estimation of MS progression, announcing a future proof of concept study with 30 patients for its validation. Since the target area was clearly determined, the first QNI step was considered satisfied.

The remaining 83 studies were based on data from large clinical trials, University hospitals or publicly available sources (see second columns of Table 3, Table 4, Table 5).

### 3.4 Automatic detection and segmentation

Many different automatic methods were developed for lesion detection and segmentation. Other studies used automatic methods, such as *k*-nearest neighbour (Fartaria et al., 2015, Todea et al., 2023, Steenwijk et al., 2013), Support Vector Machines (SVMs) (Abdullah et al., 2012, Opbroek et al., 2014, Elsebely et al., 2021, Roy et al., 2013, Hosseinipanah et al., 2019, Khotanlou and Afrasiabi, 2012, Kuwazuru et al., 2011), Markov random fields (Schmidt et al., 2011, Harmouche et al., 2014, Galimzianova et al., 2017, Subbanna et al., 2015), random forest (Geremia et al., 2011, Elliott et al., 2013, Dwyer et al., 2019), or ad hoc intensity-based algorithms (Tran et al., 2022, Cavedo et al., 2022, Brune et al., 2020, Tomas-Fernandez and Warfield, 2015, Freire and Ferrari, 2016, Mechrez et al., 2016, Meier et al., 2017, Jain et al., 2015, Sweeney et al., 2014, Egger et al., 2016, Cabezas et al., 2014, Valcarcel et al., 2020, Ong et al., 2012).

The high-level category of deep neural networks was predominant, where convolutional neural networks (CNNs) as U-Nets were most represented (see fourth column of Table 3, Table 4, Table 5). Basaran et al. (2022) adopted nnU-Net (Isensee et al., 2021), a method that automatically configures pre-processing steps, architecture, training and post-processing to better adapt to dataset properties and available hardware.

In Tripoliti et al. (2019) no details were disclosed about their automatic method and, as mentioned in Section 3.3, the reference dataset was not described. As a consequence, this conference paper did not fulfill the second QNI step.

Longitudinal methods (i.e., assessing changes in lesions' number and volume across two or more time points) adopt different approaches compared to cross-sectional methods (i.e., those using images acquired at a single time point). In fact, the evaluation of follow-up scans presents challenges, such as the one related to image registration—if patient positioning is not consistent—, and the one concerning the required pre-processing steps to account for variations in image acquisition between scans. Moreover, new lesions in follow-up scans are usually small and there is currently no threshold defining a significant lesion enlargement. To overcome these challenges, different approaches have been proposed to date such as the one proposed by Salem et al. (2022)—using a cascade of two FCNN’s to refine possible misclassifications—or the one suggested by Sepahvand et al. (2020), where an attention mechanism based on image subtraction between two timepoints was applied to help a U-Net differentiating between anatomical and artifactual change.

### 3.5 Data quality check and pre-processing

Data quality check, if mentioned, consisted of the removal of null slices (Ghosal et al., 2020, Kumar et al., 2019, Alijamaat et al., 2021, Rondinella et al., 2023), control of the scanning protocol and a thorough visual inspection (Schmidt et al., 2019). In Cavedo et al. (2022), before computing MRI analysis, a quality check of MRI parameters is performed to verify that the parameters align with those recommended. An image quality assessment was also explored in Valencia et al. (2022), through the median absolute error and the structural similarity index. Other metrics, such as lesion conspicuity, SNR (signal to noise ratio), contrast to noise ratio, and variance of the Laplacian were selected in Arnold et al. (2022). Narayana et al. (2018) used the automated pipeline validated in Narayana et al. (2013) to check headers and the SNR of DICOM images.

A more careful approach was developed in Rakic et al. (2021), dealing with T1-w and FLAIR modalities of 159 MS patients from multiple centers and scanners. In order to preserve robustness and minimize data bias, the authors followed a carefully designed protocol: the stratification of training, validation, and test set was obtained in a way to equally represent all data characteristics, such as screening site, scanner model, magnetic field strength, scan quality, slice thickness.

In Todea et al. (2023), two experts performed an image quality assessment (SNR, artifacts, contrast, good registration between time points) and a longitudinal analysis was evaluated on the whole dataset and on images with the same quality score. The same concept was applied to images obtained with a 1.5T and 3T scanner. On the other hand, in Combès et al. (2021), data with lower quality were intentionally not excluded from the study to mimic a real-world scenario.

Most studies include the following data pre-processing steps: bias field inhomogeneities correction, intensity normalization, skull stripping, denoising, resampling, and co-registration in the case of multiple input modalities.

### 3.6 Quantitative reports

**Fig. 3:**
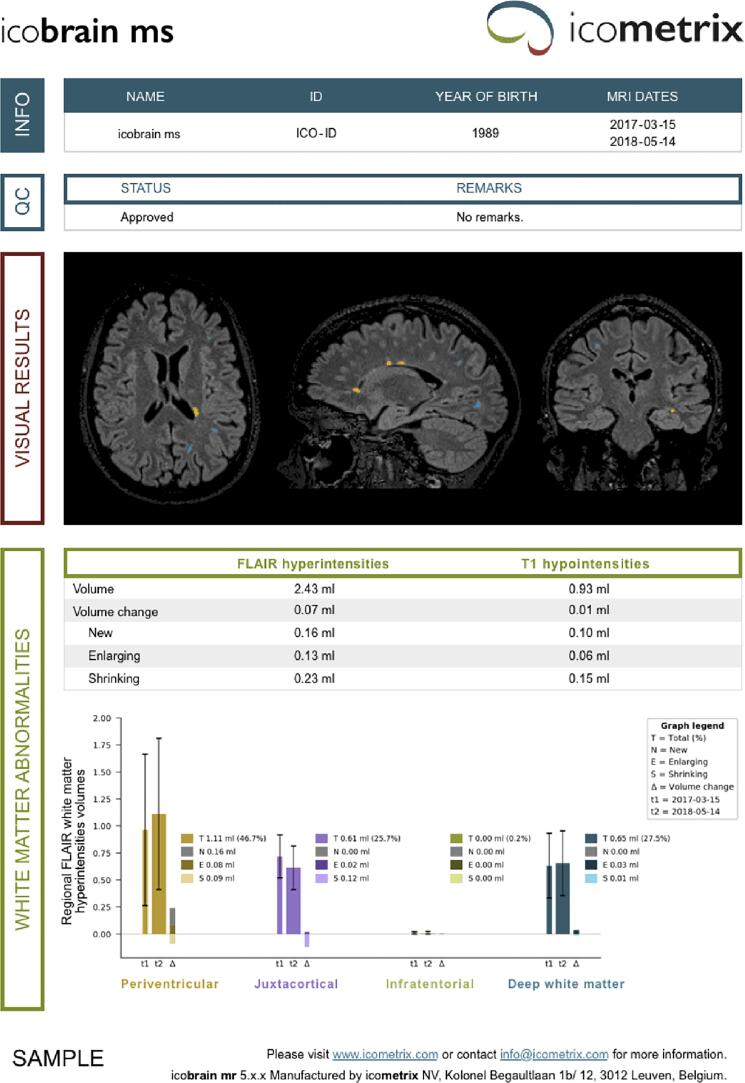
Report example published in Van Hecke et al. (2021) (a) and Brune et al. (2020) (b).

The results presented in 148 studies did not provide radiologists with a summary report. Combès et al., 2021, Brune et al., 2020, Tripoliti et al., 2019, Thakur et al., 2022, Van Hecke et al., 2021 explicitly explored the use of the developed tool to assist radiologists in generating a quantitative report, providing information such as the number, the volume and the location of lesions. Two examples are reported in Fig. 3.

In Cavedo et al. (2022), the authors presented a report with detection scores and the overlay of predictions on original images, while Yildirim and Dandil, 2021a generated a similar documentation in a web-based user interface tested by two radiologists.

In Bilello et al. (2013), the generated report contained new (or enlarging) and resolved (or improving) lesions detected, their specific location and the cerebral hemisphere involved.

### 3.7 Technical validation

The commonly explored technical evaluation metrics were those required to participate in the international contests (Maier-Hein et al., 2022):

1. Overlap-based metrics, such as Dice score coefficient (DSC), sensitivity (recall), specificity, precision, accuracy, lesion-wise true positive rate (TPR) and false positive rate (FPR), the absolute volume difference between ground truth and predicted segmentation;
2. Surface-based metrics, such as the average symmetric surface distance.

The lesion annotation through consensus was improved in the latest challenges: the available ground truth (GT) masks are more reliable in terms of inter-observer variability, providing higher quality GT to train and evaluate the models. An exhaustive list of adopted metrics is reported in the sixth columns of Table 3, Table 4, Table 5.

The latest reviews (Diaz-Hurtado et al., 2022; Commowick et al., 2023) report satisfactory and already close to human rater performances for many detection/segmentation automatic methods. However, as also mentioned in Commowick et al. (2023), there are currently little data related to the integration and use of those methods in clinical routine, especially in relation to the quantification of the uncertainty of their predictions in clinical practice.

### 3.8 Clinical validation

Combès et al. (2021) proposed a pre-use validation of their tool involving clinicians. The authors assessed the impact of the segmentation tool on experts’ performances as follows: three experts were asked to annotate a point near each lesion’s center (for 48 patients) with and without the help of the automatic tool (referred to as phases one and two). The number of marked lesions and time spent during the procedure were recorded in both cases. All experts were exhorted to conduct this experiment in situations similar to clinical practice. In particular, they were explicitly instructed to spend a reading time comparable to that of clinical routine. A few days prior to the first phase, each expert followed a short training session to get acquainted with the tool.

This experiment was evaluated through several metrics and compared between the two phases, such as the number of detected lesions (by each rater and overall), the average patient-wise number of lesions detected by experts (compared between phases using a paired t-test), or the pooled inter-expert standard deviation associated to the number of detected lesions.

In addition, the impact on routine clinical practice was assessed on six patients, with and without the tool (the two phases were two weeks apart): the experts measured the time needed from loading and reading MRI in hospital Picture Archiving and Communication Systems (PACS) to generating a radiology report. Patients were categorized in the report as showing “no activity”, “1 lesion” or “>1 lesion” with respect to baseline. Time spent to perform radiological readings for each of the three experts and each of the two settings were summarized, and the mean times elapsed in the two settings were tested for equality using a paired t-test.

A post-experiment interview was conducted to ask experts whether they were satisfied with the tool’s level of information and performance.

In Van Hecke et al. (2021), lesion segmentations were compared with the assessment of two raters, one experienced radiologist and one assistant neurologist. The experiment consisted of marking and counting MS lesions on images from 10 patients. The two raters independently assessed all images, which were shuffled and presented first as original scans, then with automatic lesion annotations. The reporting time was recorded, and the agreement between the counts reported by the two raters with and without the tool was analysed. Moreover, a similar procedure was followed to test if the help of automatic reports might change radiological findings when assessing follow-up scans.

In Bilello et al. (2013), two neuroradiologists generated a clinical report without assistance from the CAD software. Independently, the same scans were assessed by another neuroradiologist using only the software output. In both cases, new, enlarging, resolved and improving detected lesions were compared, as well as the specified lesion location. The duration of the software-assisted pipeline was also recorded for each scan, not including the image processing time.

Yildirim and Dandil, 2021a reported having their pipeline tested by two radiologists and evaluated as an auxiliary tool for diagnosis and decision support in terms of ease of use, practicality, working speed, and automatic detection. Since no details on the modality of these tests were disclosed in the article, the fourth QNI step can not be considered fulfilled.

Similarly, Hindsholm et al. (2021) only presented a qualitative assessment of output masks by radiologists. Hence, their clinical validation does not comply with the QNI framework.

Technologists involved by Thakur et al. (2022) reported the time for manual intervention to execute the tool and the time to assess and generate a report for a single patient. However, they used these findings to compare two versions of the same software instead of evaluating advantages with respect to a manual assessment. For this reason this article did not fulfill the fourth QNI step.

### 3.9 Integration into clinical workflow

In Bilello et al. (2013), the DICOM series of all the paired examinations were available in PACS to be exported and used as inputs to the automated method. Similarly, in Tripoliti et al. (2019) the user can retrieve imaging data either from the PACS or the local disk of the computer where the automatic software is installed.

In Combès et al. (2021), once stored in the local clinical PACS, MR images were pseudonymized and securely transferred into a processing hosting (certified health data hosting provider), and new lesions were automatically segmented. Then, the processed images and corresponding segmentation maps were transferred back to PACS, which could be visualized in a dedicated web MRI viewer (using DICOM format).

Van Hecke et al. (2021) developed a platform including a web portal for healthcare professionals, volumetric brain reports, and the integration with hospitals’ PACS and electronic medical record systems.

In Thakur et al. (2022), the automated software was integrated and routinely used in clinical practice since April 2012. The images were stored in PACS and converted from DICOM to NIfTI (Neuroimaging informatics technology initiative) for processing. The authors mentioned their method needs MRI scans to be acquired at the same institution.

The integration of the tool into the clinical workflow was only partially investigated in Yildirim and Dandil, 2021a, including data compatibility and the visualisation of segmented lesions overlayed with the input image. Yet, the integration of their web-based system with a hospital electronic information system, such as PACS, was not considered. Thus, the fifth QNI step was not satisfied.

### 3.10 In-use validation

Van Hecke et al. (2021) presented and tested a care management system, including a patient mobile phone application (available on Android and iOS) and a website. A first patient’s perspective survey was conducted to understand patients’ attitude towards the app, different possible features, and their level of interest in using such application. A second survey collected information such as patients’ propensity to view MRI images on their own, or if they would be interested in knowing whether there were any changes in follow-ups (such as new lesions or brain volume loss).

### 3.11 QNI steps fulfillment

**Fig. 4:**
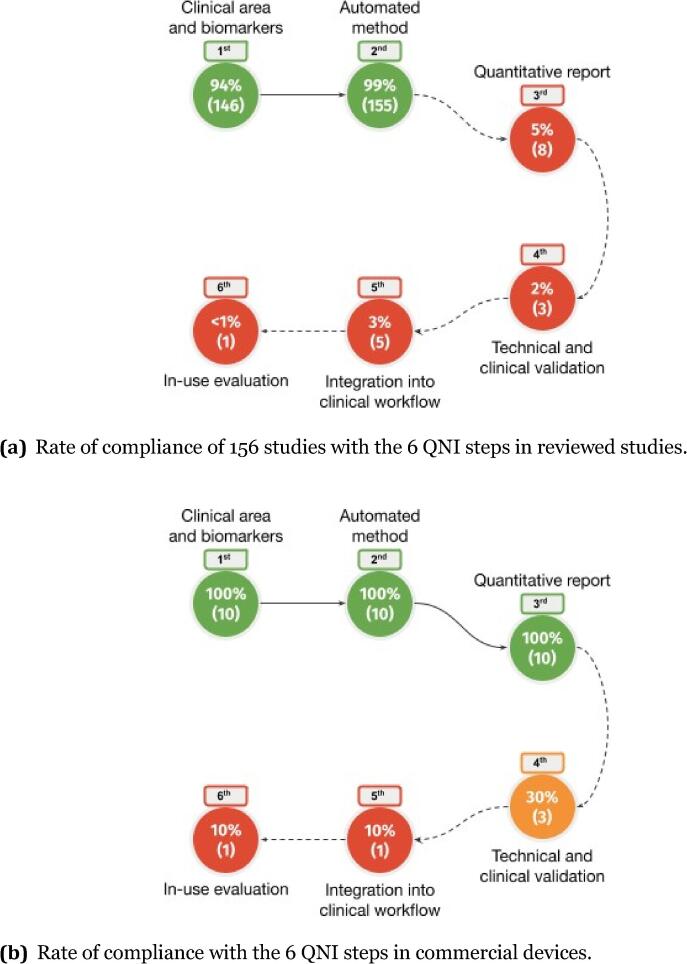
Rate of compliance with the 6 QNI steps in reviewed studies (a) and commercial devices (b).

Based on the findings presented in 156 studies, 146 comply with the first QNI step, while 155 fulfill the second. The third step is considered by eight works, three studies fully investigate the fourth and five the fifth. Only a single article explores the last QNI step. An overview of the fulfillment of QNI steps in the screened literature is presented in the road map of Fig. 4a. A similar road map can be generated from data related to 10 commercial devices screened by Mendelsohn et al. (2022), reported in Fig. 4b. A summary of the fulfilled steps is reported in the last columns of Table 3, Table 4, Table 5.

## 4 Discussion

The present systematic review exposes a considerable gap between methods’ development and the introduction of those methods into clinical practice. There are many possible cause for this gap.

A first explanation could be the difficulty to implement clinical trials: complying with clinical regulations and addressing ethical issues might result in an undesirable delay of the investigation. Participants’ insufficient knowledge about trial methods and the complexity of study protocols might also jeopardise patients’ recruitment process. The lack of trained medical personnel could represent a problem, when designing a clinical trial and even in the case of an internal clinical validation. All the above reasons are not specific to MS, meaning they could apply to many other neurological and non-neurological disorders.

Clinical integration presents, as well, some significant hurdles. To be applied in clinical practice, lesion segmentation methods should not only be integrated in the clinical workflow (i.e., be integrated in clinical PACS systems; be readily applicable to MR data that have not been preprocessed and sometimes acquired in different scanners, or with different image quality despite a consistent acquisition protocol, etc.) but also provide means to evaluate their outcome’s uncertainty and errors. Ad-hoc integration designs need to be developed considering the current clinical neuroradiological workflow as well as evaluating the reliability of those methods in a clinical routine setting, and the related clinicians’ trust in using them as clinical decision support tools. To help cover these aspects, an automatic tool could be conceived within a quality management framework for medical devices. The handling of possible failures, risk monitoring and data storage would also be addressed by following such guidelines. Data storage, management and sharing systems, such as KHEOPS (https://kheops.online/) or Flywheel (https://flywheel.io/), could be a way to deal with PACS and anonymise imaging data acquired at hospitals. Moreover, the use of a docker to execute software in an isolated and reproducible environment could help towards clinical integration. As to the real advantage of using automated methods in clinical routine, these should be carefully evaluated on site by providing means to assess errors and eventually also correct them for future evaluations, as for example could be done with uncertainty estimations/explainable AI and user-friendly interactive interfaces.

Along with this, the trade off between the economic costs of a clinical implementation and MS incidence may play an important role. In this sense, addressing medium to long-term effects (last QNI step) of the tool would be helpful. Studies should provide documentation such as:

1. periodical reports on how easily the tool could be integrated and feedback from users
2. the speed of diagnosis and failure rate, compared to pre-use cases
3. the amount of required resources, productivity, patient perception, and economic impact.

On the other hand, if a tool is not clinically adopted, its efficacy and perception could be part of the reasons. An extremely wide range of solutions with respect to the methods characteristics, inputs, and processing steps is already available and discussed in reviews, such as (Llado et al., 2012; García-Lorenzo et al., 2012; Alrabai et al., 2022; Zeng et al., 2020; Ma et al., 2022; Diaz-Hurtado et al., 2022; Commowick et al., 2023). What is actually lacking is a validation that demonstrates the advantages of automatic methods with respect to the standard procedure.

Furthermore, many of the reviewed studies have been performed on data from international challenges, which were to some extent curated and, thus, did not reflect current “real-world” clinical scenarios. Feedback from radiologists and neurologists on clinical data could help methods explore and mitigate  potential implementation biases (Vokinger et al., 2021; Varoquaux and Cheplygina, 2022). At the same time, this could change the way the tool is perceived in the clinical environment.

An additional reason may be that latest methods struggle to adapt to the heterogeneity of data acquired in clinical settings. Some recent works attempted at addressing the challenge of the use of images acquired with different contrast mechanisms and in scanners produced by different vendors and with different field strengths (Cerri et al., 2021; Billot et al., 2021). The issue represented by the different spatial resolution of clinical images, leading to variable partial volume effect during resampling, still requires ad hoc solutions and additional validation with on-site data. Also, an ad hoc integration of a method into a single institutional PACS may not generalise well in the case of a multicentric study.

Another possible motivation for the existing gap between development and clinical integration of methods could be the lack of national and international initiatives to promote their translation into clinical practice. In the current situation there is still a pronounced imbalance in favour of challenges supporting technical evaluations. Similar initiatives related to clinical validation and integration would certainly represent a boost in the implementation of solutions for MS lesion segmentation. Research focused on the integration of those methods into the clinical workflow as well as on the evaluation of their performance in a clinical routine setting might substantially help promoting their adoption and use by both neuroradiologists and neurologists.

Moreover, reducing the gap between the methods’ development and clinical translation might be highly beneficial also to improve the robustness and minimise the implementation bias of software solutions for MS lesion detection/segmentation. Ultimately, also patients would benefit from a more efficient and trustworthy process supporting disease diagnosis and monitoring of treatment effects.

## 5 Conclusions

We systematically reviewed automatic MS lesion detection and segmentation tools to assess their maturity towards clinical integration. Using the six steps of the QNI framework, we examined these quantitative tools’ development, validation, and integration level in the clinical workflow. In this review, we  focused on the required development towards clinical application of MS lesion segmentation methods, and showed that—to date—there is no consistent evidence of tools’ integration into the clinical workflow. Our work demonstrates, therefore, that there is an important gap that needs to be filled by future research in this field. In addition, the socio-economic effects and the impact on patients’ management of those tools have yet to be studied.

## Data Availability

Data will be made available on request.
